# Sulfurous thermal waters as nature-based health resources: a public health and sustainability perspective

**DOI:** 10.3389/fpubh.2026.1782645

**Published:** 2026-02-27

**Authors:** Elisabetta Ferrara, Giovanna Murmura, Giuseppe Balice, Manela Scaramuzzino, Bruna Sinjari

**Affiliations:** 1 Telematic University Leonardo Da Vinci, UNIDAV, Torrevecchia Teatina, Chieti, Italy; 2 Department of Medical, Oral and Biotechnological Sciences, School of Medicine and Health Sciences, G. D’Annunzio University of Chieti-Pescara, Chieti, Italy; 3 Thermal Medical Center of Saturnia, Grosseto, Italy

**Keywords:** chronic diseases management, non-pharmaceutic interventions, public health policy, sulfurous thermal waters, sustainable healthcare

## Abstract

The sustainability of healthcare systems has become a central public health concern as chronic diseases, rising therapeutic burden, and environmental constraints increasingly challenge service delivery models. While pharmacological treatments remain essential, complementary low-resource interventions may play a role in selected chronic conditions if framed within evidence-informed and policy-relevant care pathways. This article presents a Perspective on sulfurous balneotherapy as a potential component of sustainable models of care, using the Saturnia sulfurous thermal system as a paradigmatic and well-characterized example rather than an exceptional therapeutic resource. Drawing on concepts from public health policy, planetary health, and biological plausibility related to hydrogen sulfide exposure, the paper examines how such interventions might complement existing health services without substituting conventional medical treatments. The focus is on system-level considerations relevant to Universal Health Coverage, including therapeutic burden, patient acceptability, long-term feasibility, and integration into chronic care pathways in high-income health systems. Rather than providing a systematic review or reporting original experimental data, this Perspective aims to reframe sulfurous balneotherapy within contemporary debates on healthcare sustainability, clarify its limitations, and outline research and policy priorities needed to assess its role in equitable and resilient health systems.

## 1 Introduction

Thermal environments have long been recognized as complex natural systems in which geological, hydrological, chemical, and biological processes interact to generate resources of potential relevance for human health and well-being. Beyond their traditional therapeutic use, sulfurous springs and associated thermal muds (peloids) are increasingly investigated as nature-based systems whose properties emerge from the integration of mineral waters, clays, and biologically active components developed during maturation processes (1, 2). Among European thermal sites, Saturnia (southern Tuscany, Italy) represents a distinctive case. The spring system is characterized by the continuous emergence of sulfur-rich thermal waters at stable temperature, the formation of extensive travertine deposits, and the presence of a highly structured biotic community adapted to chemically and thermally constrained conditions. The surrounding area is recognized for its ecological value and landscape integrity, hosting habitats and species of conservation interest (3). This convergence of geodiversity and biodiversity makes Saturnia an exemplary natural laboratory for studying interactions between hydrogeochemistry, microbial life, and sedimentary processes in thermal contexts. In parallel, a growing body of literature has demonstrated that the biological fraction of thermal muds—often dominated by cyanobacteria and microalgae—plays a central role in shaping the chemical and functional properties of peloids. Studies conducted in Italian and European thermal districts have shown that microbial communities contribute to the accumulation of bioactive compounds, including lipids, fatty acids, polysaccharides, pigments, and antioxidant molecules, particularly during mud maturation (4, 5). These findings have progressively shifted the understanding of peloids from inert mineral matrices to dynamic systems in which biological processes are integral to their final composition. Specific investigations at Saturnia have confirmed the presence of a stable cyanobacteria-dominated bioglea developing in sulfurous thermal waters and sediments, with demonstrated chemical complexity and *in vitro* antioxidant activity (6). From a public health perspective, these characteristics are relevant insofar as they underpin the reproducibility and consistency of the thermal resource—prerequisites for any consideration of scalability and governance within health service planning. Such evidence situates Saturnia within a broader framework of thermal environments where microbial–mineral interactions generate compounds of potential interest for dermocosmetic, wellness, and preventive health applications, without implying direct therapeutic claims. At the same time, integrating evidence from environmental microbiology, thermal medicine, and public health highlights the need to contextualize microalgal and cyanobacterial peloids within rigorous frameworks of characterization, safety, sustainability, and governance, particularly when considering their translation into cosmetic or wellness products. This Perspective is informed by ongoing debates in public health and environmental sciences concerning the evaluation of nature-based resources beyond isolated measures of bioactivity, with increasing attention to ecological integrity, reproducibility, and long-term sustainability (7, 8). Against this background, Saturnia can be interpreted not merely as a site of historical spa use, but as an integrated natural system in which water chemistry, microbial ecology, and sedimentary processes converge to shape a distinctive thermal environment. Understanding such systems requires moving beyond reductionist approaches and adopting an explicitly interdisciplinary perspective that bridges hydrogeology, microbiology, environmental protection, and health-related applications. This Perspective aims to contribute to this discussion by proposing a conceptual framework for interpreting the Saturnia thermal system as a nature-based health resource. By situating existing evidence on sulfurous waters, bioglea, and peloids within a sustainability-oriented and ecosystem-informed view, this article identifies research gaps and outlines policy-relevant considerations for the responsible valorization of thermal environments in contemporary health and wellness contexts.

## 2 Saturnia as a hydrogeochemical–biological system

The Saturnia thermal spring system represents a well-characterized sulfurous hydrothermal environment shaped by long-term interactions between deep groundwater circulation, carbonate lithologies, and the tectonic setting of southern Tuscany. Hydrogeochemical and multi-isotopic investigations have demonstrated the homogeneity and relative isolation of the Saturnia thermal aquifer, identifying the Calcare Cavernoso formation as its primary geological host and confirming a stable chemical signature over time (4). The persistent presence of dissolved hydrogen sulfide, together with trace elements such as lithium, boron, and strontium, situates Saturnia within the broader context of central Italian geothermal systems while preserving distinct hydrochemical features (1). Notably, the long-term stability of key physical and chemical parameters—including temperature, discharge rate, and water composition—creates environmental conditions conducive to the establishment of structured and persistent microbial communities. While certain features of the Saturnia system are site-specific—such as the Calcare Cavernoso geological host, the particular travertine depositional morphology, and the local discharge characteristics—several processes observed here are likely generalizable across sulfurous thermal systems. These include hydrogen sulfide-mediated microbial selection, bioglea formation by cyanobacterial communities, and peloid maturation dynamics driven by water–rock–microbiota interactions. Distinguishing site-dependent features from transferable principles is essential for evaluating the scalability and replicability of thermal resource-based interventions in health policy contexts. These characteristics distinguish Saturnia from transient or heavily engineered thermal systems and support its interpretation as a naturally regulated hydrogeological system rather than a purely managed spa resource. Such intrinsic stability is a critical prerequisite for considering Saturnia as a reference model for studying sustained water–rock–microbiota interactions in sulfurous thermal environments. From a policy standpoint, this natural stability reduces the need for artificial standardization, potentially lowering the resource and governance burden associated with quality assurance in health-oriented applications. Sulfur-rich thermal waters provide a selective ecological niche for microorganisms capable of adapting to elevated temperatures and reduced sulfur compounds (9, 10). In the Saturnia thermal system, multiple investigations have documented the presence of a stable bioglea dominated by cyanobacteria, particularly members of the order *Oscillatoriales*, coexisting with sulfur-oxidizing bacteria and other microbial taxa (1, 2). These communities persist throughout the year, with seasonal variability primarily influencing relative abundance rather than overall taxonomic composition. From an ecological perspective, bioglea functions as a dynamic interface between thermal waters, mineral sediments, and atmospheric exposure. Microbial metabolic activity within this matrix contributes to biogeochemical cycling, primary production of organic matter, and the transformation of inorganic constituents into biologically mediated compounds (11). Such processes are increasingly recognized as central to the maturation of thermal muds and to the development of chemically complex organic fractions observed in sulfurous peloids (3). Importantly, the available evidence supports a descriptive and mechanistic interpretation of these microbial processes, without extending to claims of direct therapeutic efficacy. Within this framework, bioglea is best understood as a biologically active component of the thermal system that contributes to its chemical and ecological characteristics, rather than as a medicinal agent per se. This distinction is essential for maintaining conceptual rigor when discussing the potential relevance of microbial–mineral interactions in thermal environments for wellness or preventive health contexts.

## 3 Peloids as emergent products of natural systems

Over the last decade, the conceptualization of thermal muds has progressively shifted from that of inert mineral materials to that of complex systems shaped by coupled geological, chemical, and biological processes. Recent work has emphasized that the properties of peloids emerge from long-term interactions between mineral substrates, mineral-medicinal waters, and microbial communities, particularly during maturation phases, rather than from isolated components alone (1). Within this framework, the biological fraction—often dominated by cyanobacteria and other microorganisms—has been shown to contribute substantially to the chemical complexity of matured muds, including the accumulation of lipids, polysaccharides, pigments, and redox-active compounds (2, 12). This systems-based interpretation aligns with broader ecological evidence indicating that microbial mats and biofilms in extreme or chemically constrained environments function as dynamic interfaces that mediate biogeochemical transformations and organic matter production (9, 11). Importantly, such findings support a descriptive and mechanistic understanding of peloids as emergent natural systems, without implying direct therapeutic efficacy. From a public health perspective, framing sulfurous thermal muds within this ecological and systems-oriented paradigm allows their discussion to be anchored in biological plausibility and environmental context, rather than in efficacy-driven or pharmacological narratives. This approach is consistent with contemporary perspectives on nature-based resources, which emphasize complexity, reproducibility, and sustainability over reductionist interpretations of bioactivity (7, 8). For health systems planners, the key implication is that peloid-based interventions depend on the integrity of the natural system from which they derive—a consideration that links environmental governance directly to the sustainability of any therapeutic or preventive application.

## 4 Biological plausibility: hydrogen sulfide as a signaling molecule

Hydrogen sulfide (H_2_S) was traditionally regarded as a toxic environmental pollutant, but recent research has established it as the third gasotransmitter alongside nitric oxide and carbon monoxide, playing important roles in numerous physiological and pathological processes (13, 14). The recognition of H_2_S as an endogenous signaling molecule has prompted investigation into its potential therapeutic applications across inflammatory conditions, cardiovascular disorders, and age-associated diseases (15, 16). Studies have demonstrated that H_2_S contributes to vasodilation, anti-inflammatory responses, and cytoprotective mechanisms through multiple cellular pathways, including protein S-sulfhydration and activation of ATP-sensitive potassium channels (17). This emerging understanding of H_2_S biology provides a plausible mechanistic framework for considering sulfurous thermal waters within health-related contexts. However, translating *in vitro* and experimental findings to clinical settings remains challenging due to the narrow therapeutic window of sulfide compounds and incomplete understanding of endogenous H_2_S metabolism (14, 18). The biological plausibility provided by gasotransmitter research does not automatically validate therapeutic claims for balneotherapy, but it does suggest that exposure to sulfurous thermal environments may engage biologically meaningful pathways worthy of further investigation under controlled conditions.

## 5 Sustainability, environmental integrity, and health-oriented use

The increasing interest in thermal resources for wellness and dermocosmetic applications raises critical questions regarding sustainability, governance, and environmental protection. Saturnia is located within a landscape of high ecological value, where thermal waters, travertine formations, and associated habitats are subject to conservation frameworks and land-use constraints (3). Any form of resource valorization must therefore balance health-oriented use with ecosystem preservation. From a sustainability perspective, Saturnia offers an opportunity to reflect on how natural thermal systems can be integrated into health-related practices without compromising their ecological stability. This includes considerations of water extraction rates, sediment management, microbial community integrity, and the avoidance of excessive industrial processing that could disrupt natural maturation processes. Positioning sulfurous thermal systems within a sustainability-oriented public health framework supports a shift away from exploitative models toward stewardship-based approaches that recognize thermal environments as finite and context-dependent resources (7, 19). The WHO Regional Office for Europe has recently emphasized the potential of nature-based solutions to simultaneously address human health and environmental sustainability, advocating for approaches that generate co-benefits for both nature and well-being (8). This policy direction supports the integration of well-characterized natural resources into health promotion strategies, provided that such integration respects ecological limits and avoids overstating therapeutic claims.

## 6 Implications for public health and nature-based care models

The growing burden of chronic diseases in high-income countries has prompted increasing attention to non-pharmacological interventions that may complement conventional care while reducing therapeutic load (20, 21). Nature-based approaches, including exposure to complex natural environments, are gaining recognition as potential components of health promotion strategies, particularly for aging populations and patients with multimorbidity (22, 23). Within this framework, sulfurous thermal systems such as Saturnia offer an opportunity to explore how well-characterized natural resources might be integrated into care pathways without substituting evidence-based medical treatments. Within the continuum of wellness, prevention, and healthcare, this Perspective positions sulfurous balneotherapy primarily as a potential complementary intervention at the intersection of secondary prevention and supportive chronic care, rather than as a wellness commodity or a stand-alone therapeutic modality. This positioning implies that balneotherapy should be considered within structured care pathways for selected conditions—such as chronic musculoskeletal disorders, dermatological diseases, and post-acute rehabilitation—where existing evidence suggests biological plausibility and patient benefit, and where integration would not displace evidence-based pharmacological or surgical treatments. From a health systems perspective, balneotherapy presents several features aligned with contemporary policy priorities, namely low technological intensity, patient acceptability, potential for repeated use without cumulative toxicity, and compatibility with self-management approaches in chronic care (24, 25). However, translating these theoretical advantages into practice requires addressing key questions regarding patient selection, optimal exposure protocols, and measurable outcomes relevant to healthcare decision-makers. Integration into public health frameworks should be guided by principles of equity and accessibility, ensuring that thermal resources do not become exclusive wellness commodities but remain aligned with Universal Health Coverage objectives (26, 27). A significant barrier to integration is the heterogeneous regulatory landscape governing thermal medicine across European health systems. In some countries (e.g., Italy, France, Germany), balneotherapy is partially recognized within the national health service and subject to reimbursement under specific conditions, whereas in others it remains entirely outside the scope of public healthcare coverage. The absence of standardized reimbursement frameworks and harmonized quality criteria across jurisdictions limits the scalability of thermal interventions and creates inequities in access. Addressing these regulatory gaps is a prerequisite for any meaningful policy integration. Systematic reviews have reported improvements in signs and symptoms of psoriasis, eczematous diseases, osteoarthritis, and fibromyalgia following balneotherapy with thermal mineral waters, though methodological limitations often preclude robust conclusions (24, 28, 29). Recent evidence has also suggested potential benefits in post-COVID-19 rehabilitation, where thermal interventions demonstrated reductions in fatigue, muscle pain, and dyspnea (30). Rather than positioning sulfurous balneotherapy as a validated therapeutic intervention, this Perspective suggests that its potential lies in complementing existing services within clearly defined and rigorously evaluated care models. From a translational policy perspective, the pathway from biological plausibility to health system integration requires coordinated action at multiple governance levels. At the local and regional level, health authorities could pilot the inclusion of sulfurous balneotherapy within chronic disease management programs, particularly in territories where thermal resources are geographically accessible and where chronic musculoskeletal or dermatological conditions represent a significant burden. At the national level, health technology assessment agencies could develop evaluation frameworks specific to nature-based complementary interventions, incorporating patient-reported outcomes, cost-effectiveness analyses, and long-term sustainability metrics. At the international level, organizations such as the WHO could support the development of consensus guidelines and quality standards for thermal medicine, building on the existing policy momentum around nature-based solutions for health (8). Such a multi-level governance approach would help ensure that the integration of sulfurous balneotherapy into public health strategies is evidence-informed, equitable, and aligned with the principles of Universal Health Coverage.

## 7 Research gaps and future directions

Despite advances in hydrogeological, geochemical, and microbiological characterization, several knowledge gaps limit the evidence base needed to inform policy decisions on thermal resources in healthcare. Long-term ecological monitoring of microbial communities in sulfurous systems remains scarce, and understanding how environmental pressures such as climate variability, water extraction, and anthropogenic contamination affect bioglea composition is essential for ensuring resource reproducibility over time. Equally important is the absence of a consensus framework for characterizing thermal muds across mineral, biological, and functional dimensions; developing standardized protocols would enable meaningful comparisons across sites and support quality assurance in wellness applications. From a clinical standpoint, while biological plausibility for hydrogen sulfide-mediated effects is established, high-quality studies on sulfurous balneotherapy remain limited. Pragmatic trials embedded in real-world care settings, with patient-relevant outcomes and adequate follow-up, are needed to assess effectiveness rather than efficacy alone. More broadly, research explicitly linking ecological integrity with health outcomes is largely absent, and interdisciplinary approaches connecting environmental sciences, public health, and health economics could clarify trade-offs between resource valorization and ecosystem preservation. Addressing these gaps will require collaboration across geosciences, microbiology, clinical medicine, and policy studies. Comparative research involving multiple sulfurous thermal systems may help distinguish site-specific characteristics from generalizable processes, strengthening the foundation for evidence-informed integration of thermal resources into sustainable healthcare strategies. Such comparative efforts are particularly important for policy generalization: only by identifying which features of sulfurous systems are transferable across sites can decision-makers assess the scalability and replicability of balneotherapy-based interventions beyond individual thermal centers.

## Data Availability

The original contributions presented in the study are included in the article/supplementary material, further inquiries can be directed to the corresponding author.
